# Refining diagnostic boundaries and electroclinical profiles of Lennox–Gastaut syndrome through unsupervised clustering

**DOI:** 10.1002/epi.70403

**Published:** 2026-07-20

**Authors:** Emanuele Cerulli Irelli, Marco Perulli, Maria S. Borioni, Anna Falco, Adolfo Mazzeo, Enrico Cocchi, Maria L. Gambardella, Alessandra Morano, Michela Quintiliani, Maria Picilli, Anna T. Giallonardo, Carlo Di Bonaventura, Domenica I. Battaglia

**Affiliations:** ^1^ Department of Human Neurosciences Sapienza University Rome Italy; ^2^ Pediatric Neurology and Psychiatric Unit Fondazione Policlinico Universitario Agostino Gemelli IRCCS Rome Italy; ^3^ Department of Medical and Surgical Sciences University of Bologna Bologna Italy; ^4^ IRCCS Neuromed Pozzilli Italy

**Keywords:** cluster analysis, developmental and epileptic encephalopathy, generalized paroxysmal fast activity (GPFA), slow spike–wave discharge, subphenotype

## Abstract

**Objective:**

Lennox–Gastaut syndrome (LGS) is a developmental and epileptic encephalopathy defined by polymorphic seizures, intellectual disability (ID), and characteristic electroencephalographic (EEG) patterns. The applicability and biological validity of current electroclinical criteria remain debated. This study evaluated the concordance between clinical and electroclinical definitions of LGS and applied unsupervised clustering to identify data‐driven profiles within LGS.

**Methods:**

We retrospectively analyzed patients clinically fulfilling LGS criteria, with longitudinal electroclinical documentation and at least one sleep EEG. Patients meeting complete electroclinical criteria were compared with those meeting clinical criteria alone. Additionally, exploratory unsupervised K‐modes clustering and discriminant correspondence analysis were applied in adolescent and adult patients.

**Results:**

We included 105 patients (60.9% female), with a median age of 24 years (interquartile range = 16–35). Sixty‐nine patients (65.7%) met complete electroclinical criteria, yet neurodevelopmental features, seizure types, magnetic resonance imaging (MRI) findings, and treatment refractoriness were comparable to the clinically defined subgroup. K‐modes clustering (*n* = 97 patients) identified three data‐driven electroclinical profiles. Cluster 1 comprised patients with the earliest seizure onset, profound intellectual and motor impairment, EEG background slowing, lower prevalence of generalized paroxysmal fast activity (GPFA), highest prevalence of structural etiologies, and lowest likelihood of meeting complete electroclinical criteria for LGS. Among clusters more frequently fulfilling complete electroclinical criteria, Cluster 2 represented an early onset profile with higher rates of ID, EEG background slowing, and high prevalence of polymorphic seizures. Cluster 3 included comparatively preserved patients, characterized by later onset, milder neurodevelopmental impairment, and higher frequency of normal MRI. Despite similar clinical severity, Cluster 1 patients had undergone fewer antiseizure medication trials.

**Significance:**

Exploratory data‐driven clustering identified clinically relevant electroclinical profiles that may help refine phenotypic stratification within clinically defined LGS. These included a severe GPFA‐underrepresented profile and two GPFA‐positive phenotypes differing in developmental severity, seizure profile, and age at onset.

**Key points**

- Patients meeting complete electroclinical criteria showed broadly similar phenotypes to those with clinically defined LGS.
- Nevertheless, multidimensional data‐driven clustering identified three distinct phenotypic profiles across the LGS spectrum.
- A severe GPFA‐underrepresented profile was characterized by the earliest age at onset and marked neurodevelopmental impairment.
- The two GPFA‐positive profiles differed substantially in developmental severity, seizure patterns, and age at onset.
- Lower exposure to antiseizure medications in the GPFA‐underrepresented cluster may indicate a gap between strict electroclinical classification and treatment access in LGS.

## 1 INTRODUCTION

Lennox–Gastaut syndrome (LGS) is a severe developmental and epileptic encephalopathy (DEE) characterized by multiple seizure types, intellectual disability (ID), and distinctive electroencephalographic (EEG) patterns, including slow spike‐and‐wave discharges (SSWD) and generalized paroxysmal fast activity (GPFA).^1^ Despite these well‐described clinical and EEG features, the taxonomy of LGS remains debated, largely due to its profound etiological heterogeneity.^2^, ^3^ Since its first descriptions, nearly all recognized epilepsy etiologies—including structural, genetic, metabolic, and infectious causes—have been reported in patients with LGS.^3^

Current diagnostic criteria, recently formalized by the International League Against Epilepsy (ILAE) taskforce, integrate clinical features—including polymorphic seizure history (tonic seizures plus at least one additional seizure type) with onset before 18 years and associated mild‐to‐profound ID—and EEG criteria, requiring documentation of SSWD and GPFA during the patient's clinical course.^4^ Although these criteria provide a valuable framework, their applicability has been questioned. LGS patients may show evolution in seizure types and EEG patterns over time; for example, SSWD may disappear in adulthood, and sleep EEGs—needed to detect hallmark patterns—are often unavailable in routine clinical practice, particularly in older patients. Moreover, in real‐world cohorts, a significant proportion of patients diagnosed clinically with LGS may not meet all formal ILAE EEG criteria, reflecting possible variability in recording practices and phenotype expression.^5^

With this background, data‐driven approaches may represent a valuable strategy to critically reassess the current boundaries and phenotypic heterogeneity of LGS as a diagnostic entity. Unsupervised analytical methods allow patterns to emerge directly from multidimensional data without imposing a priori assumptions, thereby offering the possibility to identify natural groupings of patients and offering an empirical framework to assess the clinical validity of existing criteria.^6^, ^7^, ^8^

In this study, we evaluated a cohort of deeply phenotyped patients fulfilling clinically accepted criteria for LGS to determine the prevalence of complete electroclinical LGS, meeting both clinical and EEG criteria, according to current ILAE definitions. Additionally, using unsupervised clustering on a comprehensive dataset encompassing clinical, EEG, and neuroimaging variables, we aimed to investigate whether a data‐driven phenotypic approach could identify distinct clinical profiles among patients with clinically defined LGS.

## 2 MATERIALS AND METHODS

### 2.1 Study participants

This multicenter retrospective cohort study was reported and conducted in accordance with the Strengthening the Reporting of Observational Studies in Epidemiology guidelines at the epilepsy outpatient service of Sapienza University, Rome, Italy, and at the Epilepsy Unit of IRCCS Gemelli, Rome, Italy. The study was approved by the relevant institutional ethics committee (CET Lazio Area 1 and CET Lazio Area 3), and written informed consent was obtained from all participants or their legal representatives, as appropriate.

We retrospectively evaluated patients who were followed at these centers between January 1, 2020 and July 31, 2025. Patients were included if they fulfilled clinically accepted diagnostic criteria for LGS,^4^ without requiring mandatory EEG criteria. Accordingly, the following study inclusion criteria were applied: (1) a diagnosis of epileptic encephalopathy or DEE, (2) a history of tonic seizures plus at least one additional seizure type, (3) history of mild to profound ID, and (4) age at onset < 18 years. Additionally, to ensure a detailed electroclinical characterization of patients, we also applied the following inclusion criteria: availability of at least 1 h of sleep EEG, obtained through either ambulatory or in‐patient recording; and initiation of follow‐up at one of the participating epilepsy centers, or—if patients were initially managed at another institution—availability of complete and longitudinal clinical documentation from epilepsy onset.

### 2.2 Clinical data collection and EEG assessment

Clinical charts were systematically reviewed to collect demographic data, family history of epilepsy, history of febrile seizures, age at onset of first afebrile seizure, seizure types throughout the epilepsy course, occurrence of status epilepticus, magnetic resonance imaging (MRI) findings, genetic data, epilepsy etiology, psychiatric comorbidities (including use of psychiatric medications), and antiseizure medication (ASM) history.

The presence and severity of ID were determined based on standardized neuropsychological assessments and/or detailed clinical documentation. The severity of motor impairment was evaluated using the Gross Motor Function Classification System (GMFCS), a validated five‐level ordinal scale originally developed for children with cerebral palsy and widely applied in populations with neurodevelopmental disorders.^9^

Standard and sleep EEG recordings were independently reviewed by two experienced neurophysiologists (A.Ma., A.T.G., M.Pe., D.I.B.), blinded to the phenotype of the patients, at each recruiting center. Discrepancies between the two neurophysiologists were resolved through joint review and consensus. The following EEG features were assessed: (1) background activity; (2) presence and characteristics of SSWD; (3) presence of focal or multifocal epileptiform abnormalities; and (4) presence of GPFA, defined as generalized rhythmic discharges in the 10–30‐Hz range with a minimum duration of .5 s.^10^

Following EEG review, patients meeting both clinical and EEG criteria for LGS were identified and compared with patients fulfilling clinical criteria only.

All available brain MRI scans (available in 99/105 patients) were reviewed by dedicated neuroradiologists with expertise in epilepsy at each center and subsequently reviewed by experienced epileptologists (A.Ma., C.D.B., D.I.B., M.Q.), all blinded to clinical outcomes. MRI findings were initially classified as normal or abnormal based on the presence of structural abnormalities considered potentially relevant. When abnormalities were identified, lesions were characterized according to anatomical location (cortical, subcortical, or midline) and distribution pattern. Lesion distribution was categorized as unilobar, multilobar (defined as involvement of more than one lobe, either unilaterally or bilaterally in an asymmetric pattern), or diffuse (defined as bilateral and symmetric involvement).

Seizure outcome variables included the maximum duration of seizure remission achieved at any time during the disease course—categorized as none (<7 days), weeks (≥7–<30 days), or months (≥30 days)—and the presence of seizure clusters at the most recent evaluation. To ensure a standardized and reproducible definition, seizure clusters were defined as the occurrence of at least two seizures within a 2‐h interval during the last year of follow‐up.^11^

### 2.3 Statistical analysis

The K‐modes algorithm, which is specifically designed for categorical data, was employed as unsupervised clustering method. The analysis was restricted to adolescent and adult patients (≥10 years of age) to avoid inclusion of individuals with limited follow‐up, in whom longitudinal electroclinical features could not be reliably assessed. The optimal number of clusters was determined using the cost (dissimilarity) function and the average silhouette width metrics (see Supporting Information—eMethods for a detailed explanation of clustering methods).^12^

Clustering was performed using a set of clinically and neurophysiologically relevant variables encompassing seizure semiology, EEG features, neurodevelopmental severity, and comorbidities. Specifically, the following variables were included: presence of a normal MRI; history of SSWD; history of GPFA; slowing of background activity (<7 Hz) at last follow‐up EEG; severe or profound ID; infantile (≤2 years) onset of epilepsy; severe motor impairment, defined as GMFCS score ≥ 4; history of antidepressant and/or antipsychotic use; and history of atypical absences, focal seizures, atonic seizures, and/or epileptic spasms.

To visualize phenotypic structure and characterize multivariate relationships among clinical features, discriminant correspondence analysis (DiCA) was performed.^13^ This dimensionality reduction method for categorical data uses a barycentric projection to maximize separation between predefined groups and generate a low‐dimensional factorial representation of both individuals and variables. It enabled visualization of the main clinical dimensions driving cluster separation and identification of variables most strongly associated with each cluster.

Clusters were also compared using chi‐squared tests for categorical variables and independent‐samples *t*‐tests or Mann–Whitney *U*‐tests for continuous variables, depending on the normality of residuals.

All statistical analyses were performed using R software (version 4.2.3, R Foundation for Statistical Computing). Two‐sided *p*‐values < .05 were considered statistically significant.

## 3 RESULTS

### 3.1 General characteristics of study population

A total of 143 patients meeting the clinically defined criteria were initially identified. Of these, 38 patients (median age = 41 years, range = 24–60 years) were excluded because a sleep EEG was not available for review. The reasons for unavailable sleep EEG data were as follows: limited feasibility of sleep EEG recording due to behavioral comorbidities in 26 of 38 patients (68.4%); sleep EEG performed at another center before transition of care but not available for blinded review in two of 38 patients (5.3%); institutionalization or residence in another region, resulting in logistical barriers to performing the test in six of 38 patients (15.8%), and failure to fall asleep despite an attempted sleep EEG recording in four of 38 patients (10.5%).

Therefore, the final study population comprised 105 patients (60.9% female), with a median age of 24 years (interquartile range [IQR] = 16–35, range = 3–62 years) and a median age at first afebrile seizure onset of 6 months (IQR = 2–24, range = 0–192 months). Among the included patients, 97 of 105 (92.4%) were adolescents or adults (age at last follow‐up ≥ 10 years), and 74 of 105 (70.5%) were adults (≥18 years old).

The most common etiology in the cohort was structural, present in 37 of 105 patients (35.2%), followed by genetic in 22 patients (20.9%), a combination of structural and genetic in 22 (21.0%), infectious in six (5.6%), and unknown in 18 (17.1%). A detailed list of genetic etiologies is provided in eTable S1 in the Appendix, along with detailed information regarding genetic testing in the present cohort.

### 3.2 Characteristics of patients meeting LGS classification according to 2022 EEG and clinical criteria

Review of available EEG data, including sleep EEG and 24‐h ambulatory recordings, revealed variable expression of hallmark features during the entire disease course: SSWD were absent in 17 of 105 patients (16.2%), GPFA was absent in 32 of 105 patients (30.5%), and both features were absent in 13 of 105 patients (12.4%). Conversely, 69 of 105 patients (65.7%) exhibited both SSWD and GPFA and were classified as complete electroclinical LGS according to the 2022 ILAE criteria.

Electroclinical and MRI characteristics of patients meeting the complete electroclinical definition were subsequently compared with clinically defined LGS patients. No significant differences were observed with respect to age at seizure onset, degree of neurodevelopmental and motor impairment, MRI abnormalities, seizure types at onset or at last follow‐up, or seizure burden. A higher lifetime prevalence of atonic seizures was observed in the complete electroclinical LGS group (44.9% vs. 25.0%, *p* = .046), alongside a nonsignificant trend toward a higher frequency of atypical absences (59.4% vs. 41.7%, *p* = .08). Additionally, a trend toward a lower number of ASMs used over the disease course was noted in clinically defined LGS compared with the complete electroclinical LGS group (*p* = .06). A detailed comparison of the two groups is fully summarized in Table 1. Electroclinical characteristics of patients who were <10 years of age at last follow‐up are shown in eTable S2 in the Appendix.

**TABLE 1:** Characteristics of patients meeting the complete electroclinical criteria of LGS versus clinical‐only criteria.

	Complete electroclinical LGS, *n* = 69	Clinically defined LGS, *n* = 36	*p*
Age at last follow‐up, years, median (IQR)	23 (16–40)	25.5 (17–32)	.74
Female sex, *n* (%)	45 (65.2)	19 (52.8)	.21
Comorbidities, *n* (%)			
Severe intellectual disability	57 (82.6)	33 (91.7)	.21
GMFCS 4–5 motor impairment	37 (53.6)	20 (55.6)	.85
MRI features, *n* (%)			
Normal MRI	24 (36.4)	9 (27.3)	.37
Unilobar MRI involvement	9 (13.6)	1 (3.0)	.01
Multilobar or diffuse MRI involvement	33 (50.0)	23 (69.7)	.06
Cortical findings	30 (43.5)	18 (50.0)	.52
Subcortical findings	31 (44.9)	22 (61.1)	.11
Midline findings	17 (24.6)	12 (33.3)	.34
Etiology, *n* (%)			
Genetic	12 (17.4)	11 (30.6)	.12
Structural^a^	30 (43.5)	12 (33.3)	.31
Structural and genetic	13 (18.8)	9 (25)	.46
Unknown	14 (20.3)	4 (11.1)	.24
Seizure phenotype			
Infantile seizure onset, *n* (%)	54 (78.3)	30 (83.3)	.54
Age at seizure onset, months, median (IQR)	7 (3–24)	6 (1–15)	.27
Polymorphic seizures at last visit, *n* (%)	50 (72.5)	26 (72.2)	.98
Spasms, *n* (%)	39 (62.9)	25 (71.4)	.39
Atonic seizures, *n* (%)	31 (44.9)	9 (25.0)	.046
Atypical absences, *n* (%)	41 (59.4)	15 (41.7)	.08
Focal seizures, *n* (%)	39 (56.5)	21 (58.3)	.86
Tonic–clonic seizures, *n* (%)	54 (78.3)	26 (72.2)	.49
Myoclonic seizures, *n* (%)	27 (39.1)	13 (36.1)	.76
EEG findings, *n* (%)			
GPFA	69 (100)	4 (11.1)	<.001
Slow EEG background	50 (72.5)	32 (88.9)	.053
SSWD	69 (100)	19 (52.8)	<.001
Management			
Drug treatment for psychiatric comorbidities, *n* (%)^b^	11 (15.9)	4 (11.1)	.50
VNS, *n* (%)	17 (24.6)	7 (19.4)	.55
Epilepsy surgery, *n* (%)	1 (1.5)	0 (.0)	.56
ASM tried during follow‐up, mean (SD)	9.96 (3.86)	8.47 (3.62)	.06
Outcomes, *n* (%)			
Seizure clusters at last visit	24 (34.8)	7 (20.0)	.12
Longest seizure‐free interval			.67
None	44 (63.8)	26 (72.2)	
Weeks	14 (20.3)	6 (16.7)	
Months	9 (13.0)	4 (11.1)	
Mortality	5 (7.2)	2 (5.6)	.74

Abbreviations: ASM, antiseizure medication; EEG, electroencephalographic; GMFCS, Gross Motor Function Classification System; GPFA, generalized paroxysmal fast activity; IQR, interquartile range; LGS, Lennox–Gastaut syndrome; MRI, magnetic resonance imaging; SSWD, slow spike–wave discharges; VNS, vagus nerve stimulation.

^a^ Infectious etiologies were included under structural etiologies in this table.

^b^ Refers to current or previous use of antidepressants or antipsychotics.

### 3.3 Cluster analysis

K‐modes cluster analysis in adolescent and adult patients (*n* = 97) identified three distinct phenotypic profiles (*k* = 3) as the optimal solution. This model showed the highest average silhouette width among the tested solutions (.28), supporting the best internal cluster separation, and differed significantly from randomized null distributions (*p* < .001). A detailed evaluation of validation metrics is provided in the Supporting Information—eResults and eFigure S1.

Cluster 1 (*n* = 41) had the lowest median age at last follow‐up (21 years, IQR = 17–28), Cluster 2 (*n* = 42) the highest (35 years, IQR = 22–45), and Cluster 3 (*n* = 14) intermediate values (23.5 years, IQR = 18–34.5; Kruskal–Wallis test, *p* < .001). Comparison of clinical, MRI, and EEG characteristics revealed significant differences between clusters (see Table 2 for full details of the cluster differences).

**TABLE 2:** Comparison of electroclinical and MRI characteristics among identified clusters.

Characteristic	Cluster 1, *n* = 41	Cluster 2, *n* = 42	Cluster 3, *n* = 14	*p*
Age at last follow‐up, years, median (IQR)	21 (17–28)	35 (22–45)	23.5 (18–34.5)	<.001
Female sex, *n* (%)	27 (65.9)	25 (59.5)	8 (57.1)	.78
Comorbidities, *n* (%)				
Severe intellectual disability	39 (95.1)	39 (92.9)	5 (35.7)	<.001
GMFCS 4–5 motor impairment	35 (85.4)	14 (34.1)	0 (0)	<.001
MRI features, *n* (%)				
Normal MRI	10 (25.6)	12 (30)	7 (50)	.23
Unilobar MRI involvement	4 (9.8)	4 (9.5)	2 (14.3)	.87
Multilobar or diffuse MRI involvement	24 (58.5)	22 (52.4)	5 (35.7)	.34
Cortical findings	19 (48.7)	21 (52.5)	5 (35.7)	.556
Subcortical findings	25 (64.1)	20 (50)	4 (28.6)	.067
Midline findings	13 (33.3)	10 (25)	2 (14.3)	.363
Etiology, *n* (%)				
Genetic	10 (24.4)	7 (16.7)	3 (21.4)	.68
Structural^a^	22 (53.7)	12 (28.6)	4 (28.6)	.04
Structural and genetic	7 (17.1)	12 (28.6)	2 (14.3)	.34
Unknown	2 (4.9)	11 (26.2)	5 (35.7)	.009
Seizure phenotype				
Infantile seizure onset, *n* (%)	36 (87.8)	37 (88.1)	3 (21.4)	<.001
Age at seizure onset, months, median (IQR)	5 (1–12)	9 (3–16.5)	60 (36–93)	<.001
Polymorphic seizures at last visit, *n* (%)	25 (61)	36 (85.7)	8 (57.1)	.02
Spasms, *n* (%)	34 (82.9)	30 (71.4)	0 (0)	<.001
Atonic seizures, *n* (%)	4 (9.8)	30 (71.4)	5 (35.7)	<.001
Atypical absences, *n* (%)	8 (19.5)	39 (92.9)	8 (57.1)	<.001
Focal seizures, *n* (%)	10 (24.4)	39 (92.9)	8 (57.1)	<.001
Tonic–clonic seizures, *n* (%)	31 (75.6)	32 (76.2)	10 (71.4)	.94
Myoclonic seizures, *n* (%)	10 (24.4)	20 (47.6)	7 (50)	.06
EEG findings, *n* (%)				
GPFA	18 (43.9)	34 (81)	14 (100)	<.001
Slow EEG background	38 (92.7)	35 (83.3)	2 (14.3)	<.001
SSWD	32 (78)	36 (85.7)	13 (92.9)	.38
Management				
Drug treatment for psychiatric comorbidities, *n* (%)^b^	1 (2.4)	10 (23.8)	4 (28.6)	.009
VNS, *n* (%)	7 (17.1)	8 (19)	6 (42.9)	.11
Epilepsy surgery, *n* (%)	2 (4.9)	3 (7.1)	1 (7.1)	.90
ASM tried during follow‐up, mean (SD)	8.29 (3.5)	11.3 (3.8)	10.9 (4.4)	.002
Outcomes, *n* (%)				
Seizure clusters at last visit	12 (29.3)	13 (31.7)	3 (21.4)	.77
Longest seizure‐free interval				<.001
None	36 (87.8)	18 (42.9)	8 (57.1)	
Weeks	3 (7.3)	14 (33.3)	3 (21.4)	
Months	2 (4.9)	10 (23.8)	3 (21.4)	
Mortality	4 (9.8)	1 (2.4)	1 (7.1)	.37

Abbreviations: ASM, antiseizure medication; EEG, electroencephalographic; GMFCS, Gross Motor Function Classification System; GPFA, generalized paroxysmal fast activity; IQR, interquartile range; MRI, magnetic resonance imaging; SSWD, slow spike‐and‐wave discharges; VNS, vagus nerve stimulation.

^a^ Infectious etiologies were included under structural etiologies in this table.

^b^ Refers to current or previous use of antidepressants or antipsychotics.

Cluster 1 exhibited the most severe neurodevelopmental profile, with the highest prevalence of GMFCS 4–5 motor impairment (85.4%), severe ID (95.1%), and EEG background slowing (92.7%). Structurally, this group also showed a high rate of multifocal or diffuse MRI involvement (58.5%), similar to Cluster 2. Subcortical abnormalities were more frequent than in the other clusters, whereas the prevalence of cortical lesions was comparable to Cluster 2. GPFA, atypical absences, and atonic seizures were significantly less common compared with other clusters. Despite high treatment refractoriness and the highest recorded mortality rate (9.8%), these patients had been exposed to a lower cumulative number of ASMs. In terms of etiology, Cluster 1 had the highest proportion of structural causes compared with the other clusters (53% vs. 28.6% vs. 28.6%, *p* = .04).

Clusters 2 and 3 predominantly represented complete electroclinical LGS phenotypes but differed in severity and clinical features. Cluster 2 corresponded to a severe LGS phenotype, with high prevalence of infantile seizure onset (88.1%) and severe ID (92.9%), although motor impairment (34.1%) was less pronounced than in Cluster 1. This group exhibited the highest frequency of polymorphic seizures at last follow‐up (85.7%), along with a high prevalence of focal seizures (92.9%), atypical absences (92.9%), and atonic seizures (71.4%) throughout the disease course. Neurophysiologically, this profile showed both GPFA (81%) and slow EEG background (83.3%). Interestingly, Cluster 1 (82.9%) and Cluster 2 (71.4%) showed a higher prevalence of epileptic spasms during follow‐up compared with Cluster 3 (0%), as well as a higher percentage of patients with evolution from infantile epileptic spasm syndrome (IESS; 51.3% vs. 48.8% vs. 0%, respectively, *p* = .002).

Cluster 3 represented a more preserved or “late onset” LGS phenotype, with significantly lower rate of infantile seizure onset (21.4%), the highest rate of normal MRI findings (50%), and the highest rates of GPFA (100%). Patients showed the lowest rate of severe ID (35.7%) and motor impairment (0%), and the lowest rate of EEG background slowing (14.3%). Etiology remained more frequently unknown in Cluster 3 than in the other clusters (35.7% vs. 26.2% vs. 4.9%, *p* = .009), and structural or combined structural/genetic causes tended to be less common (Cluster 1: 70.8%, Cluster 2: 57.2%, Cluster 3: 42.9%, *p* = .15). Notably, 85.7% of patients in this cluster developed LGS from epilepsy onset, compared with 48.8% of patients in Cluster 2 (*p* = .03). Finally, patients in Clusters 2 and 3 were more likely to require psychiatric therapies (23.8% and 28.6%, respectively) compared with those in Cluster 1 (2.4%).

Among patients included in the clustering analysis, *CDKL5*‐related cases were predominantly observed in Cluster 1 (5/6), whereas *TSC2* cases were mainly represented in Cluster 2 (6/7). The complete distribution of genetic etiologies across the three clusters is provided in eTable S3 in the Appendix.

When considering the proportion of patients fulfilling the complete electroclinical LGS criteria, this was significantly lower in Cluster 1 (18/41, 43.9%) compared with Cluster 2 (31/42, 73.8%) and Cluster 3 (13/14, 92.9%; *p* < .001).

To visualize the multivariate structure underlying these clusters, we performed DiCA, which showed a clear separation among clusters. The first dimension, accounting for 76.0% of the explained variance, primarily reflected neurodevelopmental and encephalopathic severity, characterized by contributions from severe ID, severe motor impairment (GMFCS = 4–5), early seizure onset, EEG background slowing, and epileptic spasms. The second dimension, explaining the remaining 24.0%, primarily captured seizure phenotype, with the highest contributions from atonic seizures, atypical absences, and focal seizures, and a more modest contribution from GPFA (Figure 1, eTable S4).

**FIGURE 1:**
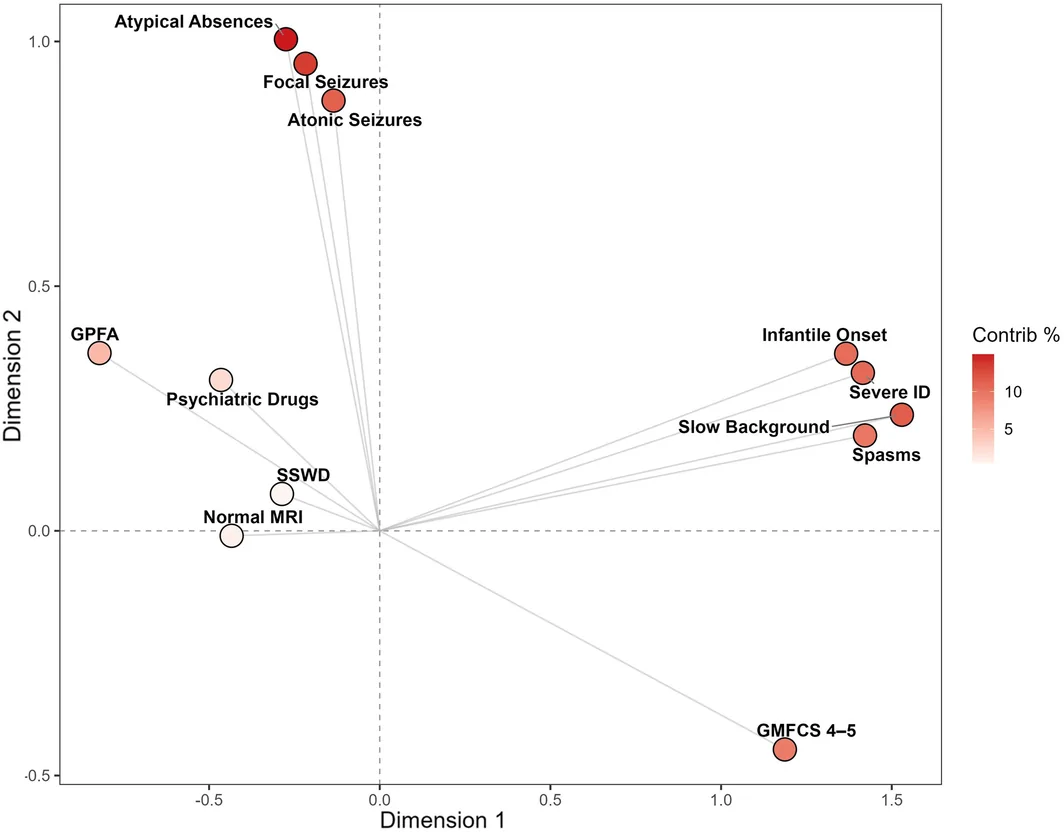
Variable contribution map from discriminant correspondence analysis. The map illustrates the distribution and multivariate relationships of clinical and electroencephalographic features across the first two dimensions. The color gradient (Contrib %) indicates the relative percentage contribution of each individual variable to the overall variance. GMFCS, Gross Motor Function Classification System; GPFA, generalized paroxysmal fast activity; ID, intellectual disability; MRI, magnetic resonance imaging; SSWD, slow spike‐and‐wave discharges. Psychiatric drugs refer to prior or current use of antidepressant or antipsychotic medications.

Geometrically, clusters occupied distinct regions of the factorial plane (Figure 2). Cluster 1, located in the lower‐right quadrant, showed high scores on the neurodevelopmental dimension and low scores on the seizure phenotype dimension, consistent with a severe encephalopathic profile marked by substantial neurodevelopmental impairment and a lower representation of the seizure‐related features loading on Dimension 2. Cluster 2, positioned in the upper part of the map, showed high scores on the seizure phenotype dimension and intermediate scores on the neurodevelopmental dimension, corresponding to a phenotype characterized by a high burden of focal seizures, atonic seizures, atypical absences, and GPFA, in association with relevant but less pronounced neurodevelopmental impairment than Cluster 1. Cluster 3 occupied the opposite end of the neurodevelopmental dimension and an intermediate position along the seizure phenotype dimension, consistent with greater preservation of motor and cognitive function compared with the other clusters and a lower expression of the polymorphic seizure phenotype (despite high representation of GPFA) compared with Cluster 2.

**FIGURE 2:**
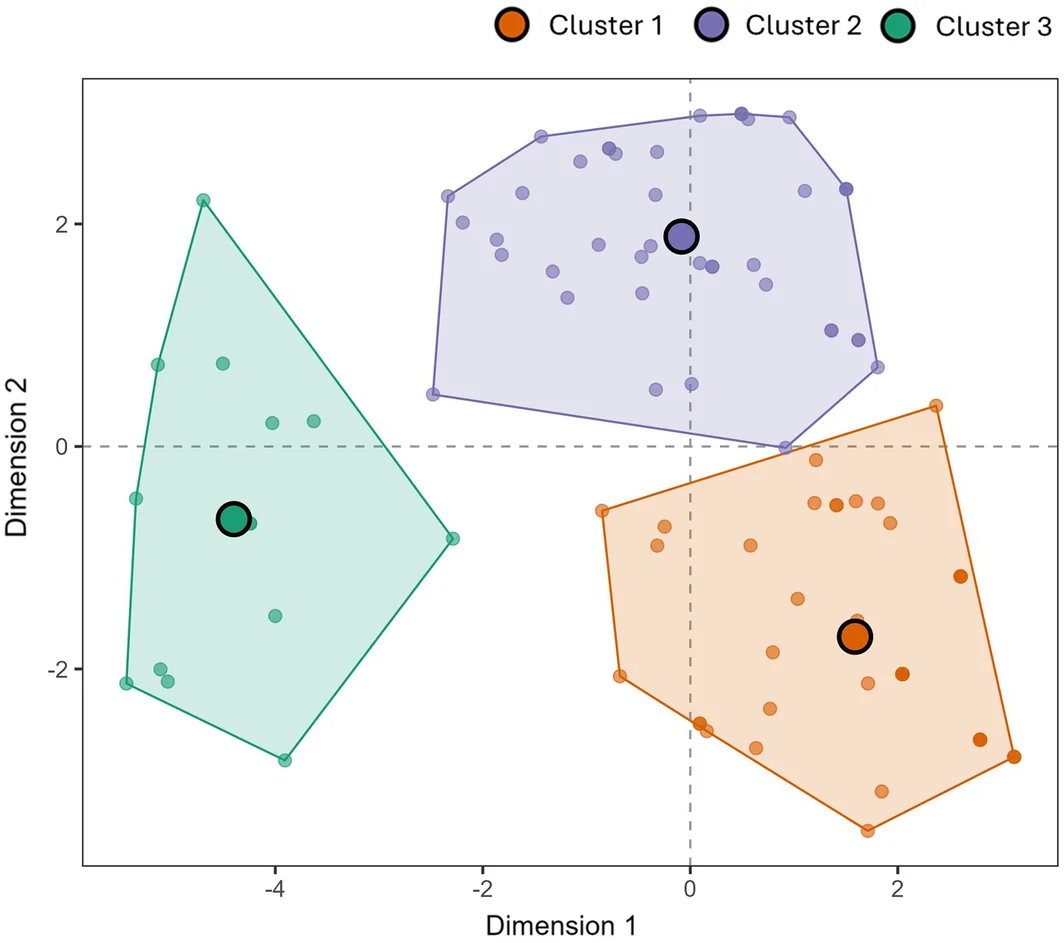
Two‐dimensional representation of clusters using discriminant correspondence analysis. Each small dot represents an individual patient positioned according to their values on the two latent dimensions derived from the clustering procedure: Dimension 1, mainly reflecting neurodevelopmental severity, and Dimension 2, mainly reflecting seizure phenotype. Patients located closer together share similar clinical and electroencephalographic characteristics, whereas patients farther apart are more dissimilar. Colors indicate the three clusters identified by the algorithm. The shaded polygons outline the distribution and boundaries of patients belonging to each cluster. The large circles represent the centroid (barycenter) of each cluster, corresponding to the average position of all patients within that subgroup.

## 4 DISCUSSION

In this deeply phenotyped cohort of patients with clinically defined LGS, we evaluated whether clinical criteria alone reliably identify patients who meet the complete electroclinical LGS definition. This issue is particularly relevant given the difficulties in obtaining retrospective EEG documentation in long‐standing disease histories,^14^, ^15^, ^16^ as well as the inconsistent application of EEG criteria for enrollment in randomized clinical trials of LGS.^17^, ^18^ Notably, only approximately two thirds of clinically defined patients fulfilled the complete electroclinical LGS criteria. However, pairwise comparisons between these groups revealed largely overlapping profiles in terms of seizure type distribution, neurodevelopmental impairment, structural MRI abnormalities, and treatment refractoriness. These findings suggest that, at the level of aggregate clinical severity and disease burden, the additional requirement of EEG criteria may not substantially modify the overall clinical characterization.

We then applied exploratory unsupervised K‐modes clustering and multivariate phenotypic analysis to investigate the internal structure of the LGS spectrum and evaluate whether distinct clinical profiles could emerge. In contrast to the limited separation observed in pairwise comparisons, this exploratory approach identified three partially overlapping phenotypic profiles that broadly aligned with current consensus definitions while capturing additional variability within the LGS spectrum.

Specifically, two clusters (i.e., Cluster 2 and 3) showed high rates of complete electroclinical LGS (73.8% and 92.9%), whereas a third cluster (i.e., Cluster 1) displayed a markedly lower prevalence of GPFA and a reduced likelihood of meeting complete criteria (43.9%).

This latter “GPFA‐underrepresented” cluster comprised patients with the earliest seizure onset, the most severe intellectual and motor impairment, marked diffuse EEG slowing, and near absence of other seizure types characteristic of LGS—such as atonic seizures and atypical absences—typically co‐occurring with tonic seizures.^15^ The combination of profound developmental dysfunction and lower prevalence of GPFA in this cluster might suggest a neural network that is either insufficiently organized or excessively disrupted to generate the synchronized high‐frequency discharges characteristic of canonical LGS. Notably, this cluster also showed the highest prevalence of structural etiologies and subcortical abnormalities, which could be relevant given the putative role of subcortical circuits in the generation of GPFA.^19^, ^20^, ^21^

Although the lower prevalence of GPFA in Cluster 1 may partly reflect neurophysiological undersampling, an inherent limitation of data collection in patients with LGS, the consistent clustering of other clinical features suggests that this group may capture a clinically severe profile within clinically defined LGS, in which the neurophysiological signature is either less evident or less consistently documented.

Interestingly, Cluster 1 also displayed the highest rate of IESS as initial epilepsy syndrome, together with Cluster 2. This finding suggests that patients with IESS may evolve either into a complete LGS phenotype, as previously described,^22^ or into a convergent phenotype characterized by tonic seizures, in which GPFA is less frequently observed. Future longitudinal studies should investigate whether these divergent trajectories are associated with distinct patterns of ASM responsiveness, or whether the underlying etiology represents the principal determinant of therapeutic outcome.

Among “GPFA‐positive” patients, Cluster 2 was characterized by a more severe LGS phenotype, including a higher prevalence of infantile onset, frequent evolution from IESS, greater rates of severe ID, higher rates of MRI structural abnormalities, and persistent polymorphic seizures at the last observation. Conversely, Cluster 3 showed a comparatively preserved phenotype, characterized by later seizure onset, milder intellectual and motor impairment, less frequent EEG background slowing, and a higher proportion of normal MRI findings. These features are consistent with prior observations linking later onset LGS to less severe developmental impairment,^23^, ^24^ and support the hypothesis that early life epileptic activity, together with structural abnormalities, may represent key determinants of disease severity and network disruption in LGS, potentially through cumulative effects on brain maturation and the establishment of widespread epileptic networks during critical developmental periods.^25^, ^26^ Notably, our clustering solution partially aligns with earlier classifications of LGS, in which “symptomatic” cases were associated with greater developmental impairment, more frequent evolution from IESS, and a higher persistence of polymorphic seizures, whereas “cryptogenic” cases more often presented with LGS from onset and exhibited a comparatively milder phenotype.^27^

An important methodological caveat is needed, however, when interpreting our findings related to the three clinical profiles observed within clinically defined LGS. The characteristics of the profiles presented should be interpreted as a descriptive representation of the multidimensional phenotypic structure generated by the variables entered into the clustering model, rather than as independent validation of discrete electroclinical subtypes.

Similarly, although the distribution of monogenic etiologies was nonrandom across clusters, with *CDKL5*‐related cases predominantly represented in Cluster 1 and TSC2‐related cases in Cluster 2, the absolute numbers of these genetic subgroups were small, precluding any definite conclusions regarding biologically driven distinctions. These observations should therefore be interpreted as exploratory findings that add an interesting descriptive layer to the three clinical profiles identified and may inform future studies.

To further investigate the structure underlying the three clinical profiles identified by the clustering analysis, we performed DiCA to explore how cluster characteristics were distributed along multivariate dimensions and to determine which variables contributed most to the explained variance. The first dimension, accounting for 76.0% of the explained variance, was mainly defined by features related to overall encephalopathic burden, including greater ID severity, severe motor impairment, early seizure onset, and EEG background slowing. By contrast, the second dimension, mainly defined by electroclinical features, including seizure types such as atonic seizures, focal seizures, and atypical absences, together with GPFA, accounted for 24.0% of the explained variance. Therefore, although DiCA confirmed a separation of the three clusters across these two dimensions, it also indicated that part of the heterogeneity captured by the clustering solution may be attributable to differences in disease severity, rather than exclusively reflecting discrete LGS profiles.^28^, ^29^, ^30^

Notably, our findings may also have implications for clinical practice. Patients in the “GPFA‐underrepresented” cluster, despite profound impairment, high disease burden, absence of even short remission periods throughout their clinical history, and elevated mortality, had undergone fewer ASM trials. A similar trend was observed among patients with clinically defined LGS compared with those meeting complete electroclinical criteria in our cohort.

This discrepancy may partly depend on factors not included in our dataset, such as differences in treatment eras, comorbidity burden, survival, caregiver preferences, tolerability, or treatment limitations related to profound disability. However, we might also speculate that this finding may reflect a potential gap in therapeutic attention,^31^ raising the issue of the distinction between strict electroclinical classification and treatment access. Contemporary clinical trials of LGS therapies typically do not require GPFA for enrollment,^17^, ^18^ and real‐world evidence suggests that ASMs approved for LGS, such as cannabidiol and fenfluramine, are effective irrespective of strict electroclinical classification or on‐label versus off‐label use.^32^, ^33^, ^34^, ^35^, ^36^ From a clinical perspective, it could be argued that patients with an LGS phenotype—regardless of GPFA status—may benefit from targeted therapies, supporting a pragmatic, phenotype‐driven treatment approach. This is in line with recent frameworks suggesting the need to move beyond rigid syndromic definitions, as current electroclinical criteria may not fully capture the biological and phenotypic diversity within the LGS spectrum.^37^ Within the same framework, emphasis was also placed on the need to identify biomarkers that may inform prognosis, guide treatment selection, and predict drug response.^37^ In this context, the identification of different phenotypic profiles within the LGS spectrum may be beneficial for the design, implementation, and interpretation of randomized clinical trials in this rare patient population.

Several limitations should be acknowledged. First, the interpretation of the “GPFA‐underrepresented” subgroup requires caution, given the known temporal evolution of EEG features in LGS.^14^, ^15^ GPFA is most reliably detected during non‐rapid eye movement sleep, and potential attenuation with age—although less consistently documented than for SSWD—has been highlighted.^14^, ^15^, ^16^ Accordingly, failure to detect GPFA may reflect longitudinal EEG changes or incomplete sampling rather than true absence, underscoring that the “GPFA‐underrepresented” cluster should be interpreted as a hypothesis‐generating electroclinical phenotype, rather than as evidence of a distinct nosological entity. Nevertheless, several methodological aspects of the present study mitigate this concern. Clinical and EEG data were reviewed longitudinally from epilepsy onset, EEG features were assessed across the disease course, and all patients underwent at least one sleep EEG. Second, although clustering provided clinically interpretable profiles, the moderate average silhouette width of .28 indicated moderate cluster separation, warranting caution against interpreting the identified profiles as discrete biological subtypes. However, this value should be interpreted in the context of categorical K‐modes clustering, for which silhouette values may be lower and less directly comparable with those obtained from K‐means clustering of continuous variables. Furthermore, the retrospective design introduces potential selection and information biases, and the predominance of adolescents and adults followed in tertiary centers may limit generalizability, particularly to individuals with milder phenotypes who transition out of specialized care. An additional source of selection bias may derive from requiring a sleep EEG for study inclusion, as this may have excluded patients in whom sleep recordings were less feasible or could not be successfully obtained, particularly adults with more severe behavioral comorbidities. Finally, the lack of systematic and uniform genetic testing across the entire cohort represents an additional limitation. This is particularly relevant for patients with apparently acquired etiologies, who were less frequently genetically tested and in whom a dual etiology could not always be excluded.

In conclusion, among patients with clinically defined LGS, only two thirds met complete electroclinical criteria, yet clinical severity and outcomes were broadly comparable across groups. Exploratory data‐driven clustering identified three phenotypic profiles that, although partially overlapping, highlight the heterogeneity within clinically defined LGS: a severe “GPFA‐underrepresented” profile and two “GPFA‐positive” profiles differing in age at onset, developmental severity, electroclinical features, and etiological background. These findings support, while also refining, current LGS criteria by identifying three data‐driven profiles that converge clinically but may present significant phenotypic differences. Finally, the lower exposure to ASMs observed in the “GPFA‐underrepresented” cluster and in patients not meeting complete electroclinical criteria, although partly attributable to unmeasured factors not included in our study, may suggest a gap between strict electroclinical classification and treatment access within current LGS frameworks.

## AUTHOR CONTRIBUTIONS

**Emanuele Cerulli Irelli:** Drafting/revision of the manuscript for content, including medical writing for content; major role in the acquisition of data; study concept or design; analysis or interpretation of data. **Marco Perulli:** Major role in the acquisition of data; study concept or design; drafting/revision of the manuscript for content, including medical writing for content. **Anna Falco:** Major role in the acquisition of data; drafting/revision of the manuscript for content, including medical writing for content. **Maria S. Borioni:** Major role in the acquisition of data; drafting/revision of the manuscript for content, including medical writing for content. **Adolfo Mazzeo:** Major role in the acquisition of data; drafting/revision of the manuscript for content, including medical writing for content. **Enrico Cocchi:** Drafting/revision of the manuscript for content, including medical writing for content; analysis or interpretation of data. **Maria L. Gambardella:** Major role in the acquisition of data; drafting/revision of the manuscript for content, including medical writing for content. **Alessandra Morano:** Major role in the acquisition of data; drafting/revision of the manuscript for content, including medical writing for content. **Michela Quintiliani:** Major role in the acquisition of data; drafting/revision of the manuscript for content, including medical writing for content. **Maria Picilli:** Major role in the acquisition of data; drafting/revision of the manuscript for content, including medical writing for content. **Anna T. Giallonardo:** Major role in the acquisition of data; drafting/revision of the manuscript for content, including medical writing for content; supervision. **Carlo Di Bonaventura:** Supervision; study concept or design; drafting/revision of the manuscript for content, including medical writing for content. **Domenica I. Battaglia:** Study concept or design; supervision; drafting/revision of the manuscript for content, including medical writing for content.

## CONFLICT OF INTEREST STATEMENT

E.C.I. has received speaker's fees from Angelini Pharma and travel support from UCB pharma, Jazz Pharmaceuticals, and Angelini Pharma outside the submitted work. M.Pe. has received honoraria from Jazz Pharmaceuticals, outside the submitted work. A.Ma. has received speaker's fees from Angelini Pharma outside the submitted work. C.D.B. has received speaker's or consultancy fees from UCB Pharma, Eisai, GW Pharmaceuticals, Jazz Pharmaceuticals, Angelini, Lusofarmaco, and Ecupharma outside the submitted work. D.I.B. has received honoraria from UCB, Jazz Pharmaceuticals, and Biocodex for participation on advisory boards and/or at conferences, outside the submitted work. None of the other authors has any conflict of interest to disclose. We confirm that we have read the Journal's position on issues involved in ethical publication and affirm that this report is consistent with those guidelines.

## Supporting information


**eFIGURE S1.** Determination of optimal cluster number.
**eTABLE S1.** Genetic etiologies (*n* = 45) identified in the cohort.
**eTABLE S2.** Electroclinical characteristics of patients with less than 10 years of age at last follow‐up (*n* = 8).
**eTABLE S3.** Genetic etiologies identified, stratified by cluster.
**eTABLE S4.** Predictor variable loadings and contributions to the discriminant dimensions.

## Data Availability

The data that support the findings of this study are available from the corresponding author upon reasonable request.
