# Supplementary material for: Refining diagnostic boundaries and electroclinical profiles of Lennox–Gastaut syndrome through unsupervised clustering
Source: Epilepsia. 2026 Jul 20;67(10):5312–24. doi: 10.1002/epi.70403 (PMC13675765; doi:10.1002/epi.70403)
Supplement: Supplementary file 1 [file EPI-67-5312-s001.docx]

**eMethods**

*Statistical analysis*

The K-modes algorithm, which is specifically designed for categorical data, was used as the unsupervised clustering method.^12^ The analysis was restricted to adolescent and adult patients (≥10 years of age) to avoid including individuals with limited follow-up, in whom longitudinal electroclinical features could not be reliably assessed. Given the very low proportion of missing data (<1% for all variables except MRI, which was missing in <5%), missing values were handled using Multivariate Imputation by Chained Equations (MICE). A single imputed dataset (m = 1) was generated using 20 iterations. This approach was chosen because of the minimal extent of missingness and to provide a complete, internally consistent dataset for clustering, while avoiding additional variability in cluster assignment that may arise from repeated imputations in an unsupervised setting.

Clustering was performed using clinically and neurophysiologically relevant categorical variables encompassing seizure semiology, EEG features, neurodevelopmental severity, and comorbidities. Specifically, the following variables were included: normal MRI; history of SSWD; history of GPFA; slowing of background activity (<7 Hz) on the most recent follow-up EEG; severe or profound intellectual disability; infantile epilepsy onset (≤2 years); severe motor impairment, defined as a GMFCS score ≥4; history of antidepressant and/or antipsychotic use; and history of atypical absences, focal seizures, atonic seizures, and epileptic spasms.

The optimal number of clusters was determined using a combined internal validation strategy. For candidate solutions across a range of K values, we examined the cost (within-cluster dissimilarity) function and the average silhouette width, and compared both metrics with their corresponding null distributions obtained from randomized data. The final choice of K was based on concordance across three considerations: (1) reduction in within-cluster dissimilarity without evidence of overfragmentation; (2) maximization of silhouette width as a measure of cluster separation; and (3) superiority over randomized solutions. Priority was given to solutions showing the highest silhouette width together with a significant improvement over random expectation.

To improve robustness against local optima, the K-modes algorithm was repeated with 1,000 independent random initializations for the selected K. The final clustering solution was defined as the run with the highest average silhouette width, with within-cluster dissimilarity used as a secondary criterion in case of ties, and this optimized solution was retained for downstream analyses.

To visualize phenotypic structure and characterize multivariate relationships among clinical features, Discriminant Correspondence Analysis (DiCA) was then performed.^13^ This method provides a low-dimensional representation of categorical data in a factorial space that maximizes separation among predefined groups, allowing simultaneous visualization of individuals, clusters, and contributing variables. This approach was used to identify the principal clinical dimensions underlying cluster separation and the variables contributing most strongly to each dimension.

Clusters were also compared using chi-square tests for categorical variables and independent-samples t-tests or Mann–Whitney U tests for continuous variables, depending on the normality of residuals.

All statistical analyses were performed using R software (version 4.2.3, R Foundation for Statistical Computing, Vienna, Austria). Two-sided p-values <0.05 were considered statistically significant.

**eResults**

K-modes cluster analysis was performed in the subgroup of adolescent and adult patients (n = 97). Cluster selection was guided by complementary internal validation metrics and comparison with a random null model. The within-cluster cost decreased progressively as the number of clusters increased, as expected, with the observed cost consistently lower than that of the null model across all tested values of k. However, no clear additional elbow was evident beyond k = 3–4, suggesting diminishing gains in within-cluster homogeneity with increasingly granular solutions. In parallel, the average silhouette width was highest at k = 3 (0.28), exceeding the corresponding null-model value and declining thereafter, with only a smaller secondary increase at k = 8. Taken together, these findings indicated that solutions with more than three clusters provided limited additional improvement in homogeneity and did not improve overall cluster separation. Accordingly, k = 3 was selected as the optimal solution, representing the best balance between parsimony, internal separation, and clinical interpretability.

**eFigure 1. Determination of Optimal Cluster Number**
*(A) Cost Analysis (Elbow Method): The total within-cluster heterogeneity (cost) is plotted against the number of clusters (k = 2 to 10). The observed cost (solid red line) is consistently lower than the random null distribution (dashed grey line), indicating non-random structure. The cost curve continues to decline substantially up to k = 4, suggesting this as a candidate solution by the elbow method.
(B) Silhouette Analysis: The Average Silhouette Width (solid blue line) was used to identify the optimal number of clusters. While the cost analysis suggested candidate solutions, including k = 4, the silhouette scores showed that k = 3 provided superior cluster separation (Silhouette = 0.279) compared with the 4-cluster solution (Silhouette = 0.248) and 2-cluster solution (Silhouette = 0.266), supporting the selection of a three-cluster model.*

| **eTable 1. Genetic etiologies (n=45) identified in the cohort.** | |
| --- | --- |
| **Genetic etiology** | **N (%)** |
| CDKL5 | 9 (20.0) |
| TSC2 | 7 (15.6) |
| PAFAH1B1 | 4 (8.9) |
| ACTL6B | 2 (4.4) |
| CHD2 | 2 (4.4) |
| STXBP1 | 2 (4.4) |
| TSC1 | 2 (4.4) |
| 15q11.1q13.2 duplication | 2 (4.4) |
| 17p13.3 deletion | 1 (2.2) |
| CACNA1E | 1 (2.2) |
| CSTB | 1 (2.2) |
| DCX | 1 (2.2) |
| GABRA3 | 1 (2.2) |
| GNAO1 | 1 (2.2) |
| LAMA2 | 1 (2.2) |
| MID2 | 1 (2.2) |
| NEDD4L | 1 (2.2) |
| PPP3CA | 1 (2.2) |
| Ring chromosome 17 | 1 (2.2) |
| SCN1A | 1 (2.2) |
| TUBB2B | 1 (2.2) |
| WNK1 | 1 (2.2) |
| Xq28 duplication | 1 (2.2) |
| Of the 105 patients, 22 had a clearly acquired etiology, of whom four underwent clinical exome sequencing based on clinical judgment. The remaining 83 patients all underwent genetic testing according to clinical practice. Patients with a suspected monogenic etiology received targeted evaluation of specific genes (e.g., TSC1, TSC2) via targeted sequencing (Sanger) and/or Fluorescence in situ Hybridization and Multiplex Ligation-dependent Probe Amplification. The remaining patients were evaluated using a next-generation sequencing (NGS) multigene panel (>300 genes) and array-CGH. If initial testing yielded negative or inconclusive results, patients subsequently underwent whole exome sequencing (WES). | |

| **eTable2. Electroclinical characteristics of patients with less than 10 years of age at last follow-up (n=8).** | |
| --- | --- |
| Age at last follow-up, y, median (IQR) | 7 (3.5-8.8) |
| Age at first afebrile seizures, m, median (IQR) | 2.5 (0.25-4) |
| Female sex, n (%) | 4 (50) |
| Comorbidities |  |
| Severe Intellectual Disability, n (%) | 8 (100) |
| GMFCS 4–5 Motor Impairment, n (%) | 8 (100) |
| MRI features |  |
| Normal MRI, n (%) | 3 (37.5) |
| Unilobar MRI involvement, n (%) | 1 (12.5) |
| Multilobar or diffuse MRI involvement, n (%) | 2 (25) |
| Cortical findings, n (%) | 3 (37.5) |
| Subcortical findings, n (%) | 4 (50) |
| Midline findings, n (%) | 4 (50) |
| Etiology |  |
| Structural, n (%) | 5 (62.5) |
| Genetic, n (%) | 4 (50) |
| Pathogenic variants identified | - CDKL5 (n=3) - PAFAH1B (n=1) |
| Seizure Phenotype |  |
| Infantile seizure onset, n (%) | 8 (100) |
| Spasms, n (%) | 8 (100) |
| Atonic Seizures, n (%) | 1 (12.5) |
| Atypical Absences, n (%) | 1 (12.5) |
| Focal Seizures, n (%) | 3 (37.5) |
| Tonic-Clonic Seizures, n (%) | 7 (87.5) |
| Myoclonic Seizures, n (%) | 3 (37.5) |
| EEG findings |  |
| GPFA, n (%) | 7 (87.5) |
| Slow EEG Background, n (%) | 7 (87.5) |
| SSWD, n (%) | 7 (87.5) |
| Management |  |
| Drug treatment for psychiatric comorbidities**, n (%) | 0 (0) |
| VNS, n (%) | 2 (25) |
| Epilepsy Surgery, n (%) | 1 (12.5) |
| ASM tried during follow-up, mean (SD) | 6.75 (2.76) |
| Outcomes |  |
| Seizure clusters at last visit, n (%) | 3 (37.5) |
| Longest seizure free interval, n (%) |  |
| None | 8 (100) |
| Mortality, n (%) | 1 (12.5) |

| **eTable 3. Genetic etiologies identified, stratified by cluster.** | | |
| --- | --- | --- |
| Cluster 1 (n=41) | Cluster 2 (n=42) | Cluster 3 (n=14) |
| - CDKL5 (n=5, 12.2%) - PAFAH1B1 (n=2, 4.9%) - 17p13.3 deletion (n=1, 2.4%) - ACTL6B (n=1, 2.4%) - CSTB (n=1, 2.4%) - GNAO1 (n=1, 2.4%) - LAMA2 (n=1, 2.4%) - Ring chromosome 17 (n=1, 2.4%) - SCN1A (n=1, 2.4%) - STXBP1 (n=1, 2.4%) - TUBB2B (n=1, 2.4%) - WNK1 (n=1, 2.4%) | - TSC2 (n=6, 14.3%) - 15q11.1q13.2 duplication (n=2, 4.8%) - TSC1 (n=2, 4.8%) - CACNA1E (n=1, 2.4%) - CDKL5 (n=1, 2.4%) - CHD2 (n=1, 2.4%) - DCX (n=1, 2.4%) - MID2 (n=1, 2.4%) - NEDD4L (n=1, 2.4%) - PPP3CA (n=1, 2.4%) - STXBP1 (n=1, 2.4%) - Xq28 duplication (n=1, 2.4%) | - ACTL6B (n=1, 7.1%) - CHD2 (n=1, 7.1%) - GABRA3 (n=1, 7.1%) - PAFAH1B1 (n=1, 7.1%) - TSC2 (n=1, 7.1%) |

| **eTable 4. Predictor variable loadings and contributions to the discriminant dimensions.** | | | | | |
| --- | --- | --- | --- | --- | --- |
| Variable | Dim 1 Position | Dim 1 Contrib (%) | Dim 2 Position | Dim 2 Contrib (%) | Total contrib (%) |
| Spasms | 1.42 | 9.24 | 0.19 | 0.55 | 9.79 |
| EEG slow background | 1.53 | 10.69 | 0.24 | 0.81 | 11.50 |
| GPFA | -0.82 | 3.08 | 0.36 | 1.91 | 4.99 |
| Severe ID | 1.42 | 9.15 | 0.32 | 1.51 | 10.66 |
| Infantile seizure onset | 1.37 | 8.53 | 0.36 | 1.90 | 10.43 |
| GMFCS IV–V | 1.19 | 6.44 | -0.45 | 2.89 | 9.33 |
| SSWD | -0.29 | 0.38 | 0.08 | 0.08 | 0.46 |
| Normal MRI | -0.43 | 0.86 | -0.01 | 0.00 | 0.86 |
| Psychiatric drugs use | 0.46 | 0.99 | -0.31 | 1.38 | 2.37 |
| Atypical absences | 0.27 | 0.35 | 1.00 | 14.60 | 14.95 |
| Atonic seizures | 0.14 | 0.08 | -0.88 | 11.19 | 11.27 |
| Focal seizures | 0.22 | 0.22 | -0.95 | 13.19 | 13.41 |
